# Neurological Signs and Symptoms in Human T-Lymphotropic Viruses 1 and 2 Infected Patients Living in the Amazon Region, Northern Brazil

**DOI:** 10.3390/v18030340

**Published:** 2026-03-10

**Authors:** Giovani Camelo do Nascimento, Lucas Thiago Ferreira Monteiro, Hemengella Karyne Alves Oliveira, Márcio Yutaka Tsukimata, Bianca Lumi Inomata da Silva, Aline Cecy Rocha Lima, Rodrigo Borges de Oliveira, Gabriel dos Santos Pereira Neto, Eduardo Leitão Maia, Ricardo Ishak, Antonio Carlos Rosário Vallinoto, Izaura Maria Vieira Cayres Vallinoto

**Affiliations:** 1 Laboratório de Virologia, Instituto de Ciências Biológicas, Universidade Federal do Pará, Belém 66.075-110, Brazil; giovani.camelo@gmail.com (G.C.d.N.); marcio.tsukimata@ics.ufpa.br (M.Y.T.); inomatabianca@gmail.com (B.L.I.d.S.); alinececy@yahoo.com (A.C.R.L.); gabrielnetenf@gmail.com (G.d.S.P.N.); rishak@ufpa.br (R.I.); vallinoto@ufpa.br (A.C.R.V.); 2 Faculdade de Medicina, Instituto de Ciências Médicas, Universidade Federal do Pará, Belém 66.075-110, Brazil; lucasthiagomonteiro@gmail.com (L.T.F.M.); hemengella.oliveira@ics.ufpa.br (H.K.A.O.); rodrigo.oliveira@ics.ufpa.br (R.B.d.O.); 3 Hospital Universitário João de Barros Barreto, Belém 66.073-000, Brazil; eduardoleitaomaia@gmail.com

**Keywords:** HTLV-1, HTLV-2, HAM, neuropathology, clinical markers

## Abstract

HTLV-1 and HTLV-2 infections are associated with various neurological manifestations, particularly HTLV-1-associated myelopathy (HAM). This descriptive, cross-sectional observational study aimed to investigate and analyze the neurological manifestations in patients treated at the Service for the Care of People Living with HTLV (Serviço de Atendimento à Pessoa Vivendo com HTLV-SAPEVH) at the Federal University of Pará. A cohort of 957 individuals underwent screening for HTLV-1/2 infection using enzyme-linked immunosorbent assay (ELISA), with seropositive samples subsequently confirmed via Western blotting or quantitative polymerase chain reaction (qPCR). HTLV-1/2 infection was confirmed in 69 individuals. Of these, fifteen individuals—diagnosed with HTLV-1 (*n* = 11) or HTLV-2 (*n* = 4) infection—who presented with neurological complaints at the first nursing consultation, were referred to a neurologist for clinical evaluation of neurological signs and symptoms. Most of the patients were female (13), ranging from 33 to 80 years of age. Neurological symptoms were present in 86.7%, and included spasticity, paraparesis, chronic pain, both motor and sensory deficits, as well as urinary disorders, predominantly affecting the thoracic spinal cord and lower limbs. Urinary symptoms were observed in 77% of symptomatic patients, often preceding other neurological signs that suggest a role as “sentinel symptoms” in the clinical screening of HTLV carriers. The results demonstrated the presence of neurological impairment in patients infected with both HTLV-1 and HTLV-2, with motor symptoms ranging from moderate to advanced. In addition, cases of cranial nerve and upper limb involvement were reported, a finding that is rarely described in the literature. The study highlights the importance of neurological assessment as early as possible in patients infected with either HTLV-1 or HTLV-2 and suggests that sphincter dysfunctions can serve as early clinical markers of future neurological impairment.

## 1. Introduction

Human T-lymphotropic virus (HTLV) is a retrovirus of the family *Retroviridae* and the genus *Deltaretrovirus* [1]. Currently, there are 4 types of HTLV [2,3,4,5,6]. HTLV-1 and HTLV-2 are the most prevalent worldwide, particularly in regions where HTLV-1 is endemic, including Japan, the Caribbean, South America, and Central Africa [7]. HTLV-1 was the first human retrovirus discovered in 1980, following its isolation from patients with cutaneous T-cell leukemia/lymphoma and adult T-cell leukemia (ATL) [2,3]. Later, a second type, known as HTLV-2, that is supposedly less pathogenic, was isolated from a patient with hairy cell leukemia [4]. HTLV-3 and HTLV-4 were isolated from hunters in Africa, although their clinical significance has not been proven [5,6].

Retroviruses are single-stranded RNA viruses that use the enzymes reverse transcriptase and integrase, respectively, to generate DNA strands and integrate them into the host cells’ genetic material in the form of a provirus [8]. This mechanism allows HTLV to persist for long periods integrated into the DNA of lymphocytes, often presenting little or no clinical manifestations, and to replicate mainly through cell-to-cell contact (virological synapse) and cellular mitosis [9,10]. The virus is transmitted through the sharing of contaminated syringes and needles, blood and tissue/organ donations, unprotected sex, and vertically, primarily via breastfeeding [7,11,12,13].

HTLV is a virus with low replicative capacity in vivo, and its proliferation depends on the mitotic expansion of infected lymphocytes [14,15]. HTLV-1 and HTLV-2 show tropism for different types of T lymphocytes. HTLV-1 infects CD4+ T lymphocytes primarily, whereas HTLV-2 exhibits tropism for CD8+ T lymphocytes [16,17,18]. The latency, oncogenicity, and immunological dysregulation enable HTLV-1 to generate a range of clinical manifestations in various human systems [19].

In addition to lymphoproliferative disease, HTLV-1 is associated with a wide range of neurological, respiratory, rheumatological, ophthalmological, fibromyalgia and dermatological diseases [20,21,22,23,24]. Nonetheless, it is suggested that most HTLV-1 carriers remain asymptomatic, with approximately 5% eventually developing severe disease related to the virus infection [25,26].

HTLV-1 and HTLV-2 share similar morphological and biological structures, but with distinct oncogenic properties [27]. To date, only HTLV-1 has been extensively studied in relation to its broad symptomatology, whereas HTLV-2 has primarily been considered an ancestral infection associated with major human migrations worldwide [28,29]. Occasionally, however, HTLV-2 has been pointed out as the cause of certain neurological, pulmonary, and rheumatological disorders [22,30,31,32,33,34].

HTLV-1 is the etiological agent of HTLV-1-associated myelopathy—HAM [20]. But some studies indicate additional neurological symptoms related to HTLV infection beyond the classical forms of HAM, which develop in only a minority of carriers [26,35]. Sensory disturbances, gait abnormalities, isolated bladder dysfunction, erectile dysfunction, dry syndrome, dysautonomia, peripheral polyneuropathy, myositis, cognitive disorders, cranial neuropathies, movement disorders, and even an amyotrophic lateral sclerosis-like syndrome have been reported in non-HAM patients [36,37]. The large spectrum of neurological symptoms associated with both HTLV-1 and HTLV-2 infections requires further in-depth studies correlating disease development with HTLV infection, especially for HTLV-2, which is also associated with neurological abnormalities [31,32,36]. Neurological alterations present in non-HAM patients become a key focus for symptomatic treatment, improving the quality of life and work capacity of the carriers [37,38]. Although there is no specific treatment or vaccine for HTLV, there is evidence that early intervention provides short-term clinical improvement in severe forms of the disease, as the initial years of HAM tend to exhibit greater intensity and progression [39,40,41,42,43].

Thus, HTLV infection has the potential to manifest varied neurological symptoms that may differ from HAM in both HTLV-1 and HTLV-2 infections, potentially resulting in symptomatic presentations. Therefore, this work aims to describe the neurological signs in people living with HTLV-1 and HTLV-2 who are attended at the Service for the Care of People Living with HTLV (Serviço de Atendimento à Pessoa Vivendo com HTLV—SAPEVH) of the Federal University of Pará (UFPA).

## 2. Materials and Methods

### 2.1. Study Design and Population Study

This study is a descriptive, cross-sectional observational analysis utilizing data from individuals gathered by the Virology Laboratory (LabVir) at UFPA. The information was collected through SAPEVH, an outpatient service that provides multidisciplinary care—including neurological assessments—for people living with HTLV (PLHTLV).

Recruitment was carried out through three main strategies: (i) engagement during health activities organized by LabVir at various sites in the metropolitan region of Belém, including primary health units, churches, and community centers; (ii) identification of individuals with confirmed diagnoses and positive serological screening results at a blood bank; and (iii) enrollment of individuals who were informed about the SAPEVH service and voluntarily presented to the laboratory for testing. Between April 2022 and March 2024, researchers assessed patients as part of this study. During that timeframe, 957 people aged 18 years or older were screened for infection, and only those diagnosed with HTLV-1 or HTLV-2 were referred to the SAPEVH outpatient clinic for further medical evaluation.

After enrollment in SAPEVH, the infected persons were offered multidisciplinary clinical care, with the objective of monitoring associated diseases, adequate treatment for PLHTLV, and supporting quality of life, including rehabilitation and dentistry. Once a neurological complaint was identified, the patients were referred for specialized medical care according to their physical limitations, either at the patient’s residence or at the SAPEVH.

### 2.2. Laboratory Analysis

Nine hundred and fifty-seven individuals provided 4 mL of peripheral blood in EDTA-treated vacuum tubes, and the presence of anti-HTLV-1/2 antibodies was investigated using an enzyme-linked immunosorbent assay (ELISA, Murex HTLV I + II, DiaSorin). Confirmation of the infection and discrimination of the viral type were performed by a line immunoassay (Inno-LIA HTLV I/II Score FUJIREBIO) and Real-Time PCR (Applied Biosystem StepOne Plus Real Time PCR), respectively.

Individuals with a confirmed diagnosis were referred to the SAPEVH to receive specific information on laboratory follow-up, medical return, and treatment, in accordance with Ministry of Health guidelines [44]. Additionally, SAPEVH offers a screening consultation with a medical professional (general practitioner) and nursing care to direct patients to specialties such as rheumatology, dermatology, and neurology. The care provided by the neurology specialist is conducted either at the LabVir headquarters at UFPA or at the patients’ residences, especially for those with mobility difficulties. After undergoing a thorough neurological investigation, the data from these patients were included in their medical records.

Out of the 957 individuals screened for HTLV infection at SAPEVH, 69 were identified as seropositive for HTLV-1/2 infection, with 56 cases confirmed as HTLV-1 (81.1%), 12 as HTLV-2 (17.4%), and 1 case classified as indeterminate (1.5%).

### 2.3. Clinical Analysis

Subsequently, the HTLV-1/2-infected participants underwent a directed anamnesis and neurological physical examination, and the degree of symptomatology was assessed. Individuals who were seronegative, not registered with SAPEVH, not screened by a SAPEVH nurse or physician, or who did not consent to participate or withdrew from the study at any stage were excluded.

The clinical information included age at symptoms onset and the evolution of the disease, underlying comorbidities, family history, occupation, use of licit or illicit drugs, blood transfusion history, breastfeeding, organ transplantation, daily life habits, sexual history, bowel rhythm, and the presence of urinary incontinence. All this information was based on the patient’s clinical complaints during the anamnesis.

The physical examination was conducted to detect clinical signs consistent with HAM, based on the criteria established by Castro-Costa et al. [20], including changes caused by damage to the thoracic spinal cord, but with minimal impact on the rest of the nervous system, that is, progressive, cumulative, and slowly evolving symptoms in the lower limbs and lumbar spine, while being absent or nonspecific in other regions, such as the cranial nerves and upper limbs.

Motor impairments were systematically classified according to three criteria: muscle tone (spasticity vs. flaccidity), degree of strength loss (paresis vs.plegia), and anatomical distribution. Assessment tools included the Medical Research Council (MRC) Scale for Muscle Strength [45]. The classification encompassed the following categories: severe motor deficit, mild motor deficit, spastic hemiparesis, spastic paraparesis, spastic paraplegia, and paraplegia with areflexia.

Initially, this investigation began with an inspection of the patient’s gait, focusing on identifying the spastic and paraparetic walk typical of HAM or, even, some degree of muscle atrophy in patients with advanced disease. Subsequently, other signs and symptoms were investigated, such as pain or paresthesias, loss of muscle strength, hypertonia, presence of clonus, hyperreflexia, a positive Babinski sign, a positive Romberg test, and a reduction in the general sensitivity of the lower limbs. In specific cases, the patient could be referred to complementary examinations, including tomography, magnetic resonance imaging, or evoked potentials, to investigate other possible diseases.

### 2.4. EIPEC-2 Scale and Data Analysis

The Evandro Chagas Research Institute Neurological Disability Scale (EIPEC-2) was used as a valuable tool for assessing and staging symptomatic patients [46]. EIPEC-2 provides a comprehensive, system-based analysis with an emphasis on objectivity from the clinician’s perspective. The scale ranges from 0 to 29 points in total, allocating up to 17 points for motor function, 3 for spasticity, 4 for sensory evaluation, and 5 for sphincter control. Higher scores correspond to greater neurological disability. Additionally, the assessment relies on objective data that depends more on the professional’s observations than on the patient’s self-report. Therefore, EIPEC-2 offers considerable utility in classifying patients as rapid, slow, or non-progressors—information that is essential in planning individualized therapeutic strategies. This stratification is particularly relevant in the early management of HAM, where symptoms tend to be more intense and accompanied by significant psychosocial impacts. Data storage and statistical analysis were performed using Microsoft Excel 2016 and Bioestat 5.0 software, respectively.

### 2.5. Ethical Aspects

This study was reviewed and approved by the Human Research Ethics Committee of the Health Sciences Institute of the Federal University of Pará (CAAE: 27290619.2.0000.0018 and CAAE: 71261523.1.0000.0018) in accordance with the guidelines of the Declaration of Helsinki. Informed consent was obtained from the patients for the publication of this article.

## 3. Results

Out of 957 people tested in the study, 69 were found to be positive for HTLV-1/2. Of these, fifteen—eleven with HTLV-1 and four with HTLV-2—had neurological complaints during their initial nursing consultation and were subsequently referred to a neurologist for further evaluation of neurological signs and symptoms (Table 1). The majority of participants were females, in that 9 (73%) women were infected with HTLV-1 and 3 (27%) with HTLV-2. Only two participants were males (13.3%), one infected with HTLV-1. The age range of the participants was between 33 and 80 years, with a mean age of 56.6 years.

Only 2 individuals (13.3%) were neurologically asymptomatic, both of whom were male. One was identified as HTLV-1 positive during prenatal screening, while the other, infected with HTLV-2, presented with respiratory symptoms. The other 13 patients (86.7%) were neurologically symptomatic, with 10 testing positive for HTLV-1 and 3 for HTLV-2.

The main neurological symptoms identified in this study involved thoracic spinal cord involvement. All of the 13 patients presented with at least one of the following symptoms affecting the thoracic and lumbar spine, as well as the lower limbs: spasticity, paraparesis, chronic pain, motor and/or sensory deficits, and urinary disturbances. Urinary symptoms were noted as an initial complaint in most HTLV-positive individuals, occurring in 10 of the 13 patients (77%). The mean age of onset of urinary symptoms was 46.0 years (38.87 years for HTLV-1 and 74.5 years for HTLV-2). These symptoms progressed from early sphincter dysfunction—typically beginning with urinary urgency and evolving into urinary incontinence or a combination of both—while in some patients the initial urinary symptom remained stable (Table 2).

Additional neurological symptoms were observed in 5 of the patients (4 with HTLV-1 and 1 with HTLV-2), including decreased trigeminal nerve sensitivity, dementia syndromes, headaches, and weakness or paresthesia in the upper limbs (Table 3). Symptoms associated with altered sensory perception, pain, and paresthesia were frequent complaints among symptomatic participants. Pain was reported by 8 out of the 13 symptomatic patients (61.5%), the majority of whom were infected with HTLV-1 (7 participants). It was most localized in the thoracic or lumbar spine and lower limbs, typically described as leg pain or cramps. Less frequently, participants also reported pain in the upper limbs and headaches.

This study categorized pain complaints by anatomical location (Table 3) into lower limb pain, upper limb pain, thoracic/lumbar pain, and headache episodes. Paresthesia occurred in 9 of the 13 symptomatic patients (69%) and was generally described in the limbs as burning sensations, electric shocks, numbness, or tingling. Another sensory alteration commonly identified during physical examination was hypoesthesia. In this context, 8 patients presented with decreased sensitivity in the lower limbs, with 5 showing unilateral and 3 bilateral involvement. Complaints involving the upper limbs were present in 2 cases, one unilateral and one bilateral. One participant infected with HTLV-1 reported tactile and painful hypoesthesia in the ophthalmic and maxillary branches of the right trigeminal nerve. The participant infected with HTLV-2 presented generalized proprioceptive sensory loss in the limbs. Information regarding the localization of hypoesthesia, type of sensory impairment, HTLV-1 or HTLV-2 infection, and the average age of participants at the time of physical examination is presented in Table 3.

Regarding motor symptomatology, 12 of the 13 symptomatic participants presented with motor impairment of the lower limbs, 9 of whom were infected with HTLV-1 and 3 with HTLV-2 (Table 4). These symptoms were commonly reported as fatigue, a sensation of heaviness, and loss of strength or movement. On physical examination, the 12 individuals with lower limb motor complaints were classified into five groups based on clinical signs identified by the neurologist: mild motor deficit, spastic hemiparesis, spastic paraparesis, spastic paraplegia, and paraplegia with areflexia. The first two categories included only one participant each, while four patients presented with spastic paraparesis, spastic paraplegia (2 patients), or paraplegia with total areflexia (4 patients). The individuals in the latter group exhibited significant hypotonia in the lower limbs, with no response to deep tendon reflex testing. Regarding the upper limbs, five participants presented with some degree of motor alteration, described as motor deficits or conditions involving spasticity or areflexia. In this assessment, one participant exhibited a mild motor deficit that did not interfere with daily activities, while another showed severe motor impairment, with complete loss of strength in the upper limbs. During the physical exam, four participants demonstrated spasticity, and one presented with areflexia.

Only the four patients diagnosed with spastic paraparesis could be evaluated using the EIPEC-2 scale for functional assessment, aiming to characterize motor, spastic, sensory, and sphincter impairments (Table 5). The mean age at which they went through the functional assessment was 60.5 years, with a mean score of 12.5 points on the scale.

## 4. Discussion

The predominance of female participants over the age of 54 in both the HTLV-1 and HTLV-2 groups in this study can be attributed to the greater efficiency of male-to-female heterosexual transmission. This dynamic contributes to increased seropositivity among older women in endemic regions, particularly as the number of sexual intercourses accumulates over a lifetime [47,48].

Another factor contributing to the predominance of older symptomatic individuals is the latency characteristic of retroviruses, which allows HTLV to remain asymptomatic for extended periods [43,49,50,51]. These long latency periods delay the clinical onset of neurological symptoms, which often emerge decades after initial infection.

The spectrum of neurological manifestations associated with HTLV-1 infection is broader than HAM, affecting both the central and peripheral nervous systems [26], and may remain undiagnosed. In the present study, among 69 HTLV-1/2 infected persons, neurological symptoms of varying severity were observed in only 13 cases. Of these, ten were attributable to HTLV-1 infection, while three involved HTLV-2, a finding that is not frequently seen and is in contrast to the anecdotal and isolated reports suggesting potential inflammatory and neurological effects in HTLV-2 carriers [22,30,31,36].

In our study, hypoesthesia emerged as a common and typically late symptom in HTLV infection, with a mean age of onset of 56.33 years. The symptom was widely distributed anatomically, initially affecting the lower limbs in eight patients (seven with HTLV-1 and one with HTLV-2), and in some cases progressing to the upper limbs or even cranial nerves. These findings are consistent with existing reports associated with HTLV-1 infection [36], but not with HTLV-2. Unilateral crural hypoesthesia was predominant at symptom onset, a presentation reminiscent of amyotrophic lateral sclerosis (ALS), as also reported by Castillo et al. [52]. Notably, one patient with advanced HAM exhibited hypoesthesia in the V1 and V2 branches of the fifth cranial nerve. The sensory loss pattern—encompassing vibratory, tactile, and painful hypoesthesia—was consistent with peripheral polyneuropathy, resulting from the asymmetric destruction induced by HTLV-associated inflammatory processes.

Motor impairment analyses further reinforced the presence of neurological symptoms in HTLV-2-infected individuals. These findings suggest that HTLV-2 may also contribute to motor deficits of considerable severity—an emerging notion that contrasts with most of the literature, which typically attributes only mild, non-motor neurological effects to HTLV-2 infection [30,31]. In this context, it is noteworthy that Neves et al. [53], in a retrospective analysis, identified that motor impairment was linked to higher risks of both hospitalization and mortality among individuals with HTLV-1. It is relevant to mention that clinical and laboratory differential diagnosis for paraparesis and paraplegia, including syphilis, Guillain-Barré syndrome, and spinal cord trauma, were excluded in this cohort.

Finally, EIPEC-2 scores highlighted the extent of motor impairment, with some patients confined to bed. Unfortunately, a longitudinal comparative analysis of progression was not feasible, as the scale was applied only once during a single neurological evaluation for four patients, which was a limitation for our study; however, a prospective clinical and laboratory study is being prepared to follow a cohort of asymptomatic and symptomatic infected HTLV-1/2 persons attending the SAPEVH.

In summary, neurological assessments revealed that individuals infected with both HTLV-1 and HTLV-2 exhibited impairments of varying severity across multiple systems. The observed symptoms were predominantly motor-related, and there was no significant difference in severity between the two viruses. It is worth mentioning that individuals infected with HTLV-2 were described, for the first time, with moderate to advanced motor sequelae. Sphincter dysfunctions were commonly observed, frequently occurring several years before the development of sensory and subsequently motor symptoms. Therefore, further studies are needed to examine more thoroughly our hypothesis that sphincter dysfunctions may serve as “sentinel symptoms,” highlighting their potential usefulness as clinical screening markers for early indications in individuals infected with HTLV-1/2. Furthermore, the occurrence of neurological manifestations associated with HTLV-2 requires further studies in larger cohorts.

The main limitations of this study include: (i) a small number of symptomatic patients, particularly those with HTLV-2 infection; (ii) possible selection bias; (iii) small number of symptomatic patients assessed by the EIPEC-2 scale (iv) the lack of an age-matched, randomized control group that is negative for HTLV-1/2; and (v) the use of a cross-sectional design, which does not allow for causal or temporal conclusions. However, these findings are still important since this is the first neurological study conducted in a cohort from northern Brazil.

Finally, our study also identified cranial nerve involvement and upper limb impairment—clinical features almost never associated with HTLV-1/2 infection. These findings highlight the need for thorough neurological assessment in all HTLV-positive individuals, regardless of type.

## Figures and Tables

**Table 1:** Distribution of HTLV-1/2 infected patients according to age, sex, and viral type.

Variable	HTLV-1/2		HTLV-1		HTLV-2	
	(*n* = 15)		(*n* = 11)		(*n* = 4)	
	*n*	%	*n*	%	*n*	%
**Sex**						
Female	13	86.7	10	91.0	3	75.0
Male	2	13.3	1	9.0	1	25.0
**Age**						
Mean ± SD	56.6 ± 15.48		54.2 ± 14.54		70.75 ± 12.17	
Range	33~80		33~80		53~80	
**Symptomatic**	13 (86.7)		10		3	
**Asymptomatic**	2 (13.3)		1		1	

**Table 2:** Distribution of HTLV-1/2-infected participants presenting sphincteric symptomatology according to viral type and symptom onset.

Symptoms	HTLV-1/2		HTLV-1		HTLV-2	
	(*n* = 13)		(*n* = 10)		(*n* = 3)	
	*n*	%	*n*	%	*n*	%
**Urinary**	10	77	8	80	2	67
Urgency	3	23	3	30	0	0
Incontinence	3	23	1	10	2	67
Urge Incontinence	3	23	3	30	0	0
Severe retention *	1	7.7	1	10	0	0
**Age of onset**						
Mean ± SD	46.0 ± 21.26		38.87 ± 17.00		74.50 ± 3.53	
Range	17~77		17~58		72~77	

* Classification based on the need for a catheter to urinate.

**Table 3:** Distribution of HTLV-1/2-infected patients according to symptoms and age at symptom onset.

Symptoms *	HTLV-1/2 (*n* = 13)		HTLV-1 (*n* = 10)		HTLV-2 (*n* = 3)	
	*n*	%	*n*	%	*n*	%
**Pain**	8	61.5	7	70	1	33.3
Lower limbs	6	46.1	5	50	1	33.3
Upper limbs	2	15.3	2	20	0	0
Lumbar/thoracic	6	46.1	5	50	1	33.3
Headache	2	15.3	2	20	0	0
**Age of onset**	Mean ± SD		Mean ± SD		Mean ± SD	
	43.62 ± 15.20		42.71 ± 16.23		53	
**Range**	22–58		22–58		53	
**Paresthesia**	9	69	8	80	1	33
Lower limbs	9	69	8	80	1	33
Upper limbs	3	23	3	30	0	0
**Age of onset**	Mean ± SD		Mean ± SD		Mean ± SD	
	43.55 ± 13.95		42.75 ± 14.69		50 ± 0	
**Range**	22–59		22–59		50	
**Symptom topography**	13	100	10	100	3	100
At or below thoracic level	5	38.4	4	40	1	33.3
Above the thoracic level	13	100	10	100	3	100
**Hypoesthesia**						
**Lower limbs**	8	61.5	7	70	1	33.3
**Unilateral:**	5	38.4	5	50	0	0
Vibratory	5	38.4	5	50	0	0
Tactile	5	38.4	5	50	0	0
Painful	4	30.7	4	40	0	0
**Bilateral:**	3	23	2	20	1	33.3
Vibratory	2	15.3	2	20	0	0
Proprioceptive	1	7.7	0	0	1	33.3
**Age**						
Mean ± SD	56.33 ± 18.63		53.28 ± 17.35		80 ± 0	
Range	33~80		33~80		80	
**Upper limbs**	2	15.3	1	10	1	33.3
**Unilateral**	1	7.7	1	10	0	0
Tactile	1	7.7	1	10	0	0
Painful	1	7.7	1	10	0	0
**Bilateral**	1	7.7	0	0	1	33.3
Proprioceptive	1	7.7	0	0	1	33.3
**Age**						
Mean ± SD	80 ± 0		80 ± 0		80 ± 0	
Range	80		80		80	
**Cranial Nerves (V)**	1	7.7	1	10	0	0
**Age**						
Mean ± SD	43 ± 0		43 ± 0		43 ± 0	
Range	43		43		43	

* The numerical values represent the number of patients who manifested the symptoms; some patients may have more than one symptom.

**Table 4:** Distribution of HTLV-1/2-infected patients according to motor impairment and age at symptom onset.

Signs and Symptoms	HTLV-1/2 (*n* = 13)		HTLV-1 (*n* = 10)		HTLV-2 (*n* = 3)	
	*n*	%	*n*	%	*n*	%
**Lower limbs**	12	92.3	9	90	3	100
Mild motor deficit	1	7.7	1	10	0	0
Spastic hemiparesis	1	7.7	1	10	0	0
Spastic paraparesis	4	30.7	3	30	1	33.3
Spastic paraplegia	2	15.3	2	20	0	0
Paraplegia with areflexia	4	30.7	2	20	2	66.6
**Age of onset**						
Mean ± SD	53.25 ± 15.49 25–80		48.22 ± 12.96 25–59		68.33 ± 13.86 53–80	
Range						
**Upper limbs**	5	38.4	4	40	1	33.3
Mild motor deficit	1	7.7	1	10	0	0
Severe motor deficit	1	7.7	0	0	1	33.3
Spasticity	4	30.7	4	40	0	0
Areflexia	1	7.7	0	0	1	33.3
**Age of onset**	57.00 ± 19.53		53.25 ± 20.36		72 ± 0	
Range	33–80		33–80		72	

**Table 5:** Average score of HTLV-1/2 infected participants, presenting spastic paraparesis.

Score	HTLV-1/2 (*n* = 4)		HTLV-1 (*n* = 3)		HTLV-2 (*n* = 1)	
	Mean ± SD	Range	Mean ± SD	Range	Mean ± SD	Range
Motor	6.25 ± 4.11	1–11	4.66 ± 3.21	1–7	4 ± 0	4
Spasticity	2.00 ± 0.81	1–3	2.33 ± 0.57	2–3	1 ± 0	1
Sensitivity	5.00 ± 2.00	2–6	4.66 ± 2.30	2–6	6 ± 0	6
Sphincter	1.00 ± 1.41	0–3	1.33 ± 1.52	0–3	0 ± 0	0
Total	12.50 ± 15.49	9–15	13.00 ± 3.46	9–15	11	11

## Data Availability

The original contributions presented in this study are included in the article. Further inquiries can be directed to the corresponding author.
